# A National Trauma Data Bank Analysis of Shark-Related Injuries on a Pediatric Scope

**DOI:** 10.7759/cureus.113034

**Published:** 2026-07-20

**Authors:** Jana Raphael, Khalil Davis, Balagangadhar Totapally, Patricio E Lau

**Affiliations:** 1 Trauma, Nicklaus Children's Hospital, Miami, USA; 2 Pediatric Critical Care, Nicklaus Children's Hospital, Miami, USA; 3 Pediatric Critical Care, Herbert Wertheim College of Medicine, Westchester, USA; 4 Pediatric Surgery, Nicklaus Children's Hospital, Miami, USA

**Keywords:** national trauma data bank, operative management, pediatric surgery, pediatric trauma, shark-related injuries

## Abstract

Background

Shark-related injuries (SRIs) in children are rare events but can result in significant morbidity due to the force and mechanism of shark bites. As coastal water use increases globally, shark-human interactions are becoming more common, creating new challenges for trauma systems and clinical management.

Methods

This retrospective observational study used the National Trauma Data Bank (NTDB) to characterize the frequency, injury patterns, severity, and clinical outcomes of pediatric SRIs in patients aged zero to 16 years from 2017 to 2021. Demographics, injury location, injury severity, hospital course, and operative interventions were analyzed.

Results

Among 520,795 pediatric trauma records, 26 cases (0.005%) were SRIs with a median age of 12 years and predominantly males (73.1%). Injuries most commonly affected the lower extremities (65.4%), followed by upper extremities (26.9%) and combined extremity injuries (7.7%). Surgical intervention was required in 25 of 26 patients, totaling 120 procedures, most commonly soft tissue operations (65.8%), followed by orthopedic (30%) and neurovascular (4.1%) procedures. The median Injury Severity Score was 5, and the median hospital length of stay was three days. Seven patients (28%) required ICU care. No in-hospital mortality occurred.

Conclusion

SRIs in children remain rare. Despite generally low injury severity with short ICU and hospital stay, nearly all cases required surgical intervention primarily for soft tissue-related intervention, followed by orthopedic procedures. These findings highlight important considerations for trauma care in pediatric SRIs.

## Introduction

Shark attacks, while relatively rare, have been increasing in U.S. coastal regions, such as Hawaii, California, and Florida, with Florida commonly recognized as the shark capital of the world [1]. Shark encounters are not only occurring more frequently but are also taking place closer to the shore, where individuals engaging in recreational water activities are typically present [2]. Scientists at the National Oceanic and Atmospheric Administration (NOAA) suggest that climate change is contributing to shark migration patterns, with species such as the hammerhead traveling farther north in search of prey [3]. Humans are also utilizing open water areas at a higher frequency in response to rising temperatures. As the utilization of marine waters for recreational activities intensifies globally, the likelihood of shark-human interactions also increases, leading to a rise in the overall number of shark attacks [1].

Shark attacks are a relatively rare presenting diagnosis in most trauma departments, which can lead to variability in clinical response and documentation. A 30-year review of the Global Shark Attack File demonstrated an overall increase in unprovoked shark bites [4]. According to the International Shark Attack File, there were 108 shark-human interactions globally in 2022, with 57 resulting in unprovoked bite wounds. As of 2023, the United States leads in shark-related attacks and injuries, with Florida consistently reporting the highest number of unprovoked bites. In fact, Florida’s 16 cases accounted for 39% of all shark-related incidents in the U.S. and 28% of unprovoked bites worldwide [5].

Despite the extreme public perception surrounding shark attacks, these injuries are less frequent and less fatal than commonly believed [6]. However, when they do occur, particularly in pediatric patients, they present unique clinical challenges due to the variability in wound patterns, risk of infection, and limited clinical experience among providers. For pediatric trauma teams, understanding the nature and management of shark-related injuries (SRIs) is essential to ensure accurate assessment, timely intervention, and better outcomes. A national descriptive analysis of these cases can help fill critical gaps in clinical knowledge, improve preparedness, and support evidence-based care in regions where shark-human interactions are becoming more common.

This study aimed to characterize the national frequency, injury patterns, injury severity, and clinical outcomes of pediatric SRIs among patients aged zero to 16 years using the National Trauma Data Bank (NTDB).

## Materials and methods

A retrospective review was conducted using the NTDB Participant Use Data File (PUF) for the years 2017 through 2021, provided by the American College of Surgeons. A waiver of IRB review was granted due to the use of de-identified data, in accordance with federal regulations.

For this analysis, a pediatric patient was defined as an individual aged zero to 16 years. The patients with SRIs were identified by using the ICD-10 codes W56.42XA (“struck by shark”), W56.41XA (“bitten by shark”), and W56.49XA (“other contact with shark”). Exclusion criteria were patients older than 16 years, records with missing key variables, including injury mechanism, procedural data, or hospital outcomes, and injuries not directly attributed to shark contact. The data extracted included patient demographics (age, sex, race, and ethnicity), injury location (upper extremity, lower extremity, or both), and hospital course details, such as Glasgow Coma Scale (GCS) at arrival, hospital admission status, length of stay (LOS), intensive care unit (ICU) admission, ICU LOS, and discharge disposition. Procedural data were categorized using ICD-10 codes and grouped into three categories: skin and soft tissue, orthopedic, and neurovascular procedures, based on anatomical location and type of intervention.

Only cases with documented operative reports or corresponding procedure codes in the NTDB were included in the procedural analysis to ensure accurate representation of injury types and treatment approaches. Descriptive statistics were used to summarize the data. Categorical variables were reported as frequencies and percentages, while continuous variables were presented as medians with interquartile ranges (IQRs). All statistical analyses were performed using SPSS version 28 (IBM Corporation, Armonk, NY). Given the descriptive nature of the study and small sample size, no inferential statistical analyses were performed.

## Results

Between January 2017 and December 2021, a total of 520,795 pediatric trauma records (ages zero to 16 years) were reported in the NTDB. Of these, 26 (0.005%) cases were identified as SRIs resulting from shark bites. None of the patients included in this review were recorded as having been injured by being struck (W56.42XA) or through other contact with a shark (W56.49XA). One patient had complete injury diagnosis data but was missing ICD-10 codes for surgical procedures, hospital course, and discharge disposition and was excluded from relevant portions of the analysis. The median age of the patients was 12 years (IQR: 10-13). The majority of the patients were male (19; 73.1%) and White (24; 92.3%).  Of the 26 patients who were attacked by sharks, seven (26.9%) sustained upper-extremity injuries, 17 (65.4%) sustained lower-extremity injuries, and two (7.7%) suffered injuries to both lower and upper extremities (Table 1). The majority of SRI patients required at least one surgical intervention, with a total of 120 procedures performed in 25 out of 26 patients. Of these, 79 (65.8%) were soft-tissue procedures. These included muscle debridement/repair (n = 35), skin/subcutaneous tissue debridement/repair (n = 40), muscle/fascia flap coverage (n = 3), and skin grafting (n = 1). Orthopedic procedures accounted for 36 interventions (30%), including bone fixation, fasciotomy, amputation, and 20 tendon/ligament repairs. Neurovascular procedures, including arterial bypass, arterial ligation, primary vascular repair, and nerve repair, were less common, representing five procedures in total (4.2%) (Tables 2, 3).   

**Table 1:** Characteristics, body region injured, hospital course, and disposition

Characteristics	
N	26
Median age, year (IQR)	12 (10-13)
Race	
White, n (%)	24 (92.3)
Other, n (%)	2 (7.7)
Ethnicity	
Hispanic, n (%)	0
Non- Hispanic, n (%)	24 (92.3)
Not known, n (%)	2 (7.7)
Sex	
Female, n (%)	7 (26.9)
Male, n (%)	19 (73.1)
Body region injured	
Upper extremity, n (%)	7 (26.9)
Lower extremity, n (%)	17 (65.4)
Both upper and lower extremity, n (%)	2 (7.7)
Hospital course	
Injury Severity Score, median (IQR)	5 (4-8)
Median Glasgow Coma Scale, median (IQR)	15 (15-15)
Pts requiring hospital admission, n	25
Average hospital LOS (days)	4
Pts requiring ICU admission, n	7
Average ICU LOS (days)	3.4
Disposition	
Mortality, n (%)	0
Discharged home/self-care, n (%)	24 (96.0%)
Discharged transferred home under care of organized home health services, n (%)	1 (4.0%)

**Table 2:** Procedures performed

Procedures	Upper extremity	Lower extremity	Total
Neurovascular	2	3	5
Orthopedic	12	24	36
Soft Tissue	25	54	79

**Table 3:** Total number of procedures by anatomic category

Procedures	Upper extremity	Lower extremity	Total
Neurovascular	2	3	5
Arterial bypass	0	0	0
Arterial ligation	0	0	0
Primary vascular repair	1	0	1
Nerve repair	1	3	4
Orthopedic	12	24	36
Bone fixation	3	10	13
Tendon/ligament repair	7	13	20
Fasciotomy	0	0	0
Amputation	1	0	1
Other	1	1	2
Soft Tissue	25	54	79
Skin/subcutaneous tissue debridement/repair	10	30	40
Muscle debridement/repair	13	22	35
Muscle/fascia flap coverage	2	1	3
Skin grafting	0	1	1

The median Injury Severity Score was 5 (IQR: 4-8). The median GCS score was 15 (IQR: 15-15). Twenty-five patients were recorded as requiring hospital admission. The median hospital length of stay was four days (IQR: 2-6). Seven (28%) of those 25 patients required an ICU admission. The median ICU length of stay (LOS) was 3.4 days (IQR: 2-4.5). Twenty-four patients (96.0%) were discharged home under self-care. One patient (4.0%) was transferred home under the care of an organized home health service. There were no mortalities during the study period (Table 1).  

## Discussion

SRIs in pediatric patients are extremely uncommon, accounting for only 0.005% of pediatric trauma cases reported in the NTDB between 2017 and 2021. These injuries often require urgent surgical intervention due to their severe mechanisms, including penetrating trauma, extensive soft tissue damage, and neurovascular compromise. In this cohort, all injuries were to the extremities, and no deaths occurred among patients who arrived at a trauma center. This study adds to the limited national literature describing pediatric shark-related trauma and highlights important considerations for trauma care despite the rarity of these injuries.

Prevalence

The rarity of pediatric SRIs in our study aligns with prior literature, including adult series, which also report a low overall incidence [7]. Although SRIs have increased in some coastal regions, potentially due to environmental changes and increased recreational water use, the prevalence among children remains low [2-3]. This difference may reflect differences in exposure to higher-risk aquatic activities between the pediatric and adult populations. Therefore, the very low prevalence observed in our analysis is consistent with national and international trends demonstrating that children represent only a small fraction of annual shark encounters.

Demographics

The demographic profile of affected children in this study, particularly with respect to age, sex, and race, may reflect exposure patterns related to recreational ocean activity. The median age was 12 years, most patients were male, and the most affected racial group was White, which may reflect behavioral and exposure-related risk factors previously described in adolescent and adult populations. The predominance of White male victims of SRIs is noted in the adult population [8]. These similarities suggest comparable exposure patterns across age groups, particularly during recreational water activities.

Location and injury characteristics

All injuries occurred to the extremities, with the lower extremities more frequently affected. The pattern is consistent with prior literature, including adult studies, suggesting that the lower extremities are more frequently targeted due to their greater exposure and movement during aquatic activity [7,9]. There were no deaths in this cohort, which may be explained by the injury pattern since an extremity injury has a higher likelihood of overall survival. 

Although rare, the clinical impacts of pediatric SRIs can be significant. Nearly all patients required hospital admission, and a total of 120 surgical procedures were performed across three major categories: soft tissue, orthopedic, and neurovascular. 

Surgical interventions

Soft tissue interventions predominated, accounting for nearly (70%) all procedures, reflecting the extensive tissue disruption that characterizes shark bite injuries and the frequent need for staged debridement and wound management [7]. Orthopedic procedures were focused on limb-salvaging interventions, including tendon and ligament repairs and bone fixation, rather than extensive amputations. Notably, the only amputation in the cohort was limited to the finger. Neurovascular procedures were comparatively uncommon, suggesting that although vascular and nerve injuries do occur, catastrophic neurovascular compromise is not as common in pediatric SRIs.

Notably, three patients required more than 10 procedures each, underscoring the severity and complexity of their injuries; however, even among these high-acuity cases, no mortalities occurred. In addition, a 13-year-old patient underwent a total of 25 procedures. Although the injuries primarily involved the upper extremities, they also sustained a torso injury requiring multiple debridements. These additional interventions did not alter the overall clinical outcome but did contribute to a prolonged PICU stay. In comparison, another patient required only a single operation, a bypass of the right ulnar artery to the right lower arm artery using autologous arterial tissue, highlighting that even highly complex vascular reconstructions can occasionally be managed with a single definitive procedure.

Outcomes

Although the Injury Severity Score (ISS) scores were low, with a high GCS, most patients required surgical intervention, and about a quarter (27%) of admitted patients required ICU observation. This is consistent with the adult literature that showed that although injury severity can be severe, they tend not to be lethal once the patients arrive at the hospital [7]. A review of unprovoked shark attacks from 2010 to 2019 found that only 6.8% of 799 attacks were fatal [8]. There were no deaths in our cohort, although it is possible that patients who died on scene were not captured by this trauma database, and therefore this is a limitation for this study. Previous adult studies have shown that patients who died from SRI died of hypovolemic shock, which highlights the importance of pre-hospital treatment and hemorrhage control. 

The predominance of soft tissue procedures, followed by orthopedic and neurovascular interventions, reflects the complex nature of SRIs in pediatric trauma. These findings are consistent with prior literature, which similarly reports high rates of nerve and vascular injuries requiring multidisciplinary surgical care [7,10]. Furthermore, 96% of patients were discharged home under self-care, suggesting favorable outcomes when appropriate care is provided. These findings align with prior adult data, which similarly reported a median ISS of 5, a high rate of surgical intervention (93%), and no in-hospital mortality among 53 patients treated at trauma centers [7]. Despite the potential for devastating injuries, including amputations and neurovascular damage, mortality from SRIs remains extremely rare, with most deaths occurring at the scene rather than in-hospital [7]. However, the infrequency of SRIs may contribute to variability in clinical response, especially in centers with limited experience managing such cases. 

Although the data presented in our study are only a portion of the total SRI cases in the United States, analysis of the NTDB enabled us to investigate a significantly larger number of SRIs than a single-center study. As a result, we were able to provide a more comprehensive summary of SRIs treated at trauma centers across the United States.

This study provides a unique and focused analysis of pediatric SRIs in the United States, offering valuable insights into injury patterns, treatment approaches, and outcomes. As shark-human interactions become more common due to environmental and behavioral factors, trauma centers, particularly in coastal regions, should be prepared to manage these complex injuries with evidence-based protocols and interdisciplinary care. Future research should aim to develop standardized guidelines for acute and long-term management of SRIs to improve preparedness and optimize patient outcomes. 

Limitations

Several limitations should be considered when interpreting these findings. As a retrospective analysis of the NTDB, this study is subject to potential coding errors, misclassification, and inaccuracies in data entry. The identification of SRIs and procedural classification relied on ICD-10 codes, which may not fully capture the clinical complexity of injuries or interventions, introducing potential misclassification bias. Additionally, the NTDB includes only patients treated at participating trauma centers, which may introduce selection bias and limit generalizability, as patients treated at non-trauma centers or those who died prior to hospital arrival are not captured. The small sample size reflects the rarity of pediatric SRIs and limits the ability to perform more robust statistical analyses or draw definitive conclusions.

The NTDB lacks detailed clinical information, including information on injury circumstances, timing of interventions, and long-term functional outcomes. As a result, this study cannot assess long-term morbidity, recovery, or quality of life following injury. These limitations should be considered when interpreting the findings, and future prospective studies or multi-institutional registries are needed to provide a more comprehensive evaluation of pediatric SRIs.

## Conclusions

Although SRIs are extremely rare in pediatric trauma, this study demonstrates that they are frequently associated with extremity injuries and a high rate of operative intervention. Among more than half a million NTDB records, only 26 cases were identified, yet many required extensive surgical interventions involving multiple specialties. In-hospital outcomes among patients treated at trauma centers were generally favorable, with no observed mortality in this cohort. These findings provide a descriptive characterization of pediatric SRIs treated at U.S. trauma centers and may help inform clinical awareness and preparedness.

## References

[1] Liang Liang, J J (2025). Shark bites consistent with recent trends, with small spike in fatalities. https://www.floridamuseum.ufl.edu/science/shark-bites-consistent-with-recent-trends-with-small-spike-in-fatalities/.

[2] Howard J (2025). Shark attacks are more common in the Atlantic Ocean. National Geographic.

[3] (2025). Tagged hammerhead shark travels widely in warm Pacific waters. https://www.fisheries.noaa.gov/news/tagged-hammerhead-shark-travels-widely-warm-pacific-waters.

[4] McPhee D (2014). Unprovoked shark bites: are they becoming more prevalent?. Coast Manag.

[5] (2025). Yearly worldwide shark attack summary. https://www.floridamuseum.ufl.edu/shark-attacks/yearly-worldwide-summary/.

[6] Macdonald C, McEntee K, Wester J (2023). Values, attitudes, and media exposure: Public perception of sharks and shark conservation in the USA. Biol Conserv.

[7] Ganske W, Sharma R, Kaminski S, Johnson A (2021). Shark-related injuries in the United States: a National Trauma Data Bank analysis. Am Surg.

[8] Scala VA, Hayashi MS, Kaneshige J, Haut ER, Ng K, Furuta S. (2026). Attacks & fatalities. Trauma Surgery & Acute Care Open.

[9] Tomberg RJ, Cachaper GA, Weingart GS (2018). Shark related injuries: a case series of emergency department patients. Am J Emerg Med.

[10] Ricci JA, Vargas CR, Singhal D, Lee BT (2016). Shark attack-related injuries: epidemiology and implications for plastic surgeons. J Plast Reconstr Aesthet Surg.

